# Antibiotic-induced gut microbiota depletion enhances glucose tolerance linked to GLP-1 signaling

**DOI:** 10.3389/fendo.2025.1684155

**Published:** 2025-11-27

**Authors:** Alexandra Kellenberger, Revati Sumukh Dewal, Alice de Wouters d’Oplinter, Andreas Sichert, Markus Heine, Marceline M. Fuh, Emma Slack, Tenagne Delessa Challa, Christian Wolfrum

**Affiliations:** 1 Laboratory of Translational Nutrition Biology, Department of Health Sciences and Technology, Institute of Food, Nutrition and Health, ETH Zurich, Schwerzenbach, Switzerland; 2 Laboratory for Mucosal Immunology, Department of Health Sciences and Technology, Institute of Food, Nutrition and Health, ETH Zurich, Zurich, Switzerland; 3 Department of Biochemistry and Molecular Cell Biology, University Medical Center Hamburg-Eppendorf, Hamburg, Germany

**Keywords:** gut microbiome, gut microbiota depletion, glucose tolerance, insulin sensitivity, GLP-1 signaling, UCP1-independent mechanism, antibiotic treatment, bile acids

## Abstract

**Introduction:**

Depletion of the gut microbiota is known to improve glucose metabolism and modify thermogenic capacity in mice. However, the underlying mechanisms remain unclear. In this study, we aimed to determine whether the browning effect observed after antibiotic treatment contributes to metabolic modifications and to investigate the potential central role of GLP-1 in enhancing glucose metabolism.

**Methods:**

Using an inducible Ucp1DTR mouse model to transiently ablate UCP1^+^ cells, we assessed glucose tolerance, cold sensitivity, and circulating GLP-1 levels following gut microbiota depletion. We additionally examined GLP-1 levels in germ-free mice. Glucose tolerance was compared to GLP1R KO mice following gut microbiota depletion. Bile acid profiling in wild-type mice treated with antibiotics identified regulated bile acids, which were subsequently tested in an *in vitro* STC-1 cell assay and *in vivo* in Cyp2c70 mice to identify potential basal GLP-1 secretion inhibitors.

**Results:**

We demonstrate that gut microbiota depletion improved glucose tolerance independent of UCP1^+^ cell presence and increased cold sensitivity. Antibiotic treatment increased circulating active GLP-1 levels within one day, and this increase was also observed in germ-free mice, supporting the suggestion that GLP-1 elevation is driven by gut microbiota depletion. The improvement in glucose tolerance was lost in GLP1R KO mice upon oral glucose ingestion. Bile acid profiling and subsequent validation led to the identification of two potential basal GLP-1 secretion inhibitors.

**Discussion:**

Our findings suggest that the metabolic improvements following gut microbiota depletion are primarily driven by GLP-1 signaling, rather than UCP1⁺ cell activation. These results highlight the complex interplay between the gut microbiome and metabolic health, offering insights into potential therapeutic targets for improving glucose metabolism through modulation of basal GLP-1 signaling.

## 1 Introduction

A balanced microbiome contributes to healthy metabolic functions, whereas dysbiosis has been linked to a range of metabolic diseases, including obesity, type 2 diabetes, and insulin resistance (1–4). The gut microbiome is highly sensitive to environmental factors, including diet, antibiotic (Abx) treatment, and stress, all of which can induce changes in microbial composition that either disrupt or improve glucose homeostasis (5–9). Recent studies have further revealed that gut microbiota influences thermogenesis and energy expenditure (10–12). Germ-free (GF) mice not only exhibit reduced adiposity and protection against diet-induced obesity but also show improved glucose metabolism (13). Similarly, Abx-induced gut microbiota depletion in lean mice leads to improved insulin sensitivity, fasting glucose, and glucose tolerance without changes in body weight, but with alterations in the luminal and serum bile acid (BA) pool as well as reduced short-chain fatty acid (SCFA) availability (14, 15). One prominent hypothesis to explain these findings is that browning of white adipose tissue (WAT) contributes to the observed metabolic improvements (12). However, different studies reported impaired thermogenesis upon gut microbiota depletion (16–18). This was further supported by showing that cold exposure alters gut microbiota composition, and transplantation of this cold-adapted microbiota to GF mice led to increased insulin sensitivity and WAT browning (16). Additionally, some studies argue that the intestinal microbiome is dispensable for adaptive thermogenesis (19, 20). These conflicting results highlight the complexity of the relationship between the gut microbiome and thermogenesis. In addition to browning, several other mechanisms have been proposed to explain the improved glucose metabolism observed in gut microbiota-depleted mice. Li et al. showed that Abx-induced gut microbiota depletion increased glucose uptake in both caecum and brown adipose tissue (BAT), independent of Uncoupling Protein 1 (UCP1) (20). Another hypothesis suggests that microbiota depletion improves glucose metabolism through the regulation of hepatic gluconeogenesis, potentially by affecting hepatic TCA cycle fluxes (19). Furthermore, a study by Zarrinpar et al. indicated that Abx-induced gut microbiota depletion in mice alters enterocyte metabolism, shifting colonocytes from using SCFAs to glucose as an energy source (15). The same study also confirmed that antibiotic-induced microbiota depletion alters whole-body BA metabolism and showed increased fasting serum total glucagon-like peptide 1 (GLP-1) levels, but the improvement in glucose tolerance could only be partially attributed to this change (15).

In this study, we aimed to clarify the mechanisms behind the improved glucose metabolism upon antibiotic-mediated gut microbiota depletion in mice. Specifically, we sought to determine whether the browning of WAT or GLP-1 contributes to the observed enhanced whole-body glucose metabolism. Furthermore, we aimed to identify the factors that increased basal GLP-1 secretion following microbiota depletion.

## 2 Materials and methods

### 2.1 Animal models and treatments

The animals used in this study were male C57BL/6N, GF, GLP-1 receptor knockout (GLP1R KO), Cyp2c70 knockout, and Ucp1Dtr2Gfp (Ucp1DTR) mice, aged 12 weeks at the start of experiments. Mice were housed in groups of two to five per individually ventilated cage under specific pathogen-free (SPF) or GF conditions on a reversed 12h dark/light cycle at room temperature (RT, 23°C), thermoneutrality (TN, 30°C), or cold (CE, 8°C) depending on the experimental setting. Mice had *ad libitum* access to a standard chow diet and water.

For UCP1^+^ cell ablation, Ucp1DTR or wild-type control mice were subcutaneously (sc.) injected with 3 doses of 100 ng Diphtheria Toxin (DT; Sigma-Aldrich, 322326) every 6h on day 0. For sustained UCP1^+^ cell deletion, one dose of 100 ng DT was injected subcutaneously twice per week. Gut microbiome depletion was induced by providing an antibiotic cocktail in sterile drinking water *ad libitum* to Ucp1DTR, wild-type, and GLP1R KO mice. The antibiotic water in the drinking bottle was refreshed twice per week, and fresh antibiotic water was prepared weekly. The antibiotic cocktail consisted of 100 µg/ml neomycin (Sigma-Aldrich, N6386), 50 µg/ml streptomycin (Sigma-Aldrich, S9137), 100 U/ml penicillin (Sigma-Aldrich, P7794), 50 µg/ml vancomycin (Sigma-Aldrich, V1130), 100 µg/ml metronidazole (Sigma-Aldrich, M1547), 1 mg/ml bacitracin (Sigma-Aldrich, 11702), 125 µg/ml ciprofloxacin (Sigma-Aldrich, 17850), and 100 µg/ml ceftazidime (Sigma-Aldrich, C3809).

The animals were euthanized by CO_2_ overdosage with a 50% flow rate of cage volume per minute. All animal experiments conducted in our lab were approved by the Cantonal Veterinary Office of Zurich, Switzerland.

### 2.2 Physiological measurements *in vivo*

Mice were fasted for 6h before measuring their baseline glucose levels (Roche, Accu-Chek Aviva). 2 g/kg body weight glucose was administered either intraperitoneally (ip.) or orally, and blood glucose levels were recorded at indicated timepoints.

During the period of temperature recording (total 6h), mice were fasted. The tip of the thermometer was dipped in Vaseline. The anal sphincter was stimulated with the tip of the thermometer, which was ultimately inserted into the anus while the mouse was restrained to avoid movement until the temperature measurement was completed.

Energy expenditure and metabolic parameters were recorded using the Phenomaster metabolic cage system (TSE system).

### 2.3 Tissue harvesting and blood sampling

Mice were sacrificed after 6h of fasting. Inguinal white adipose tissue (ingWAT), interscapular brown adipose tissue (iBAT), and the liver were snap-frozen in liquid nitrogen and stored at −80°C until further processing. The small intestinal parts (duodenum—first 8 cm; jejunum—middle; ileum—last 8 cm), caecum, and colon were dissected. The fecal content was pushed out and collected. The intestinal tissue got flushed with PBS, snap-frozen in liquid nitrogen, and stored at −80°C until further processing. The gall bladder was punctured with a 30G insulin syringe, and bile was collected in a PCR tube. For the GLP-1 time course experiment, blood was collected from the tail vein. Blood was immediately mixed with 100 µl/ml 5 mM EDTA and, if needed for GLP-1 measurement, with an additional 10 µl/ml DPP4 inhibitor (Sigma-Aldrich, DPP4-M) and 11.1 µl/ml 550 µM aprotinin (Sigma-Aldrich, A1153). This was followed by subsequent centrifugation at 8000 rcf at 4°C for 10 min, and plasma was transferred into new tubes and stored at −80°C.

### 2.4 Biochemical assays

Plasma insulin, GLP-1, glucagon, and lipopolysaccharides (LPS) were measured using the Ultra Sensitive Mouse Insulin ELISA Kit (Crystal Chem, 90080), the V-PLEX Plus GLP-1 (active) Kit (mesoscale, K1503OG), the Mouse Glucagon ELISA Kit (Crystal Chem, 81518), and the Lipopolysaccharides (LPS) ELISA Kit (abbexa, abx150357) following the manufacturer’s instructions.

### 2.5 Protein isolation and Western blot

An ingWAT or iBAT depot was homogenized in RIPA buffer (50 mM Tris/HCl pH 7.5, 150 mM NaCl, 5 mM EDTA, 1% Triton X100, 0.5% sodium deoxycholate, 10% glycerol) containing 100X protease inhibitor (Roche, 5056489001) and 100X phosphatase inhibitor (Thermo Fisher Scientific, Zug, Switzerland). The lysate was centrifuged at 4°C for 10 min at maximum speed. Total protein was determined using the Pierce BCA Protein Assay Kit (Thermo Fisher Scientific, 23225) following its instructions. 30 µg of protein was loaded onto an SDS-polyacrylamide gel (4% stacking, 10% separation density). Following separation, protein was transferred onto a 0.2 µm nitrocellulose membrane (Bio-Rad, 1620112). Blocking was done in 5% milk for 1h and subsequently incubated with the UCP1 Polyclonal Antibody (1:1000, Invitrogen, PA1-24894) in 5% milk at 4°C overnight. The HRP-linked Anti-rabbit IgG Antibody (1:4000, Cell Signaling, 7074) was used as a secondary antibody. Imaging was done with the ChemiDoc MP Imaging System (Bio-Rad).

### 2.6 mRNA isolation and gene expression analysis

mRNA isolation of liver and ingWAT was done using GENEzol (Geneaid, GZR200) following the manufacturer’s instructions. Reverse transcription of 100 ng RNA into cDNA was performed with the High-Capacity Reverse Transcription kit (Applied Biosystems, 4368813). cDNA was diluted to 5 ng/µl, and gene expression was measured with quantitative RT-PCR using the KAPA Sybr Fast kit (Roche, SFUKB) on a 384-well format Viia 7 machine (Applied Biosystems). Primer sequences are listed in **Table 1**.

**Table 1:** Murine qPCR primers used for gene expression analysis.

Gene	Species	FWD (5′ – 3′)	REV (5′ – 3′)
*Ucp1*	Mouse	CAGCCGGCTTAATGACTGGA	TGATCCCATGCAGATGGCTC
*Tbp*	Mouse	GAAGCTGCGGTACAATTCCAG	CCCCTTGTACCCTTCACCAAT

### 2.7 Metabolite profiling

SCFAs were extracted from cecal contents after homogenization in PBS using a Tissue Lyzer at maximum speed (30 Hz/s, 2.5 min). A standard solution was prepared containing the six compounds of interest, with final concentrations of 1 mM for acetate, lactate, propionate, butyrate, and succinate, and 10 mM for formate, using 50% isopropanol. Eight serial dilutions of the standard solution were made in 50% isopropanol. An internal standard solution was prepared with isotope-labeled versions of the compounds in LC-water. A master mix was prepared containing methanol, LC-water, 100 mM aniline, 50 mM EDC, 2 M HCl, and internal standard solution. For the reaction, 75 µl of the master mix was added to 25 µl of each standard/sample in a 96-well polypropylene plate (Thermo Fisher). The mixture was incubated for 30 min at 4°C, followed by quenching the reaction with 500 mM β-mercaptoethanol. The samples were incubated for an additional 30 min at room temperature before being analyzed with the LC-MS.

BAs were extracted from caecal samples using methanol containing BHT and deuterated internal standards added prior to extraction. Sample weights were recorded for data normalization after measurement. An external calibration curve, ranging from 0.0006 µM to 27 µM, was used for quantification. The analytes were separated using reversed-phase chromatography on a C18 column, with a mobile phase consisting of methanol/acetonitrile, 5 mM ammonium acetate, and 0.1% formic acid. The eluents were analyzed by MRM mass spectrometry, with measurements taken in either positive or negative electroionization mode.

### 2.8 *In vitro* experiments

STC-1 cells were kindly provided by Boehringer-Ingelheim. Cells were kept in full medium containing high-glucose (4.5 g/l) DMEM (Gibco, 41966-029) supplemented with 10% fetal bovine serum (Gibco, 10500-064) and 1% penicillin/streptomycin (Gibco, 15140-122) in a humidified cell culture incubator (37°C, 5% CO_2_). Full medium was changed every 2–3 days, and cells were split 1:5 upon reaching 70% confluency.

For GLP-1 secretion assays, cells were seeded in 24-well plates and used upon reaching 80% confluency. Cells were washed two times with prewarmed secretion buffer (138 mM NaCl, 4.5 mM KCl, 4.2 mM NaHCO_3_, 1.2 mM NaH_2_PO_4_ (2H_2_O), 2.5 mM CaCl_2_ (2H_2_O), 1.2 mM MgCl_2_ (6H_2_O), 10 mM HEPES; supplemented with fresh 0.1% fatty acid–free bovine albumin serum) and subsequently incubated in secretion buffer for 1h in the cell culture incubator. Meanwhile, a stimulation buffer containing secretion buffer supplemented with 20 µl/ml DPP4 inhibitor (Sigma Aldrich, DPP4-M) was prepared and used as a control. For positive control, 10 mM glucose was added to the stimulation buffer. For stimulation with BAs, stimulation buffer was supplemented with 30 µM of the respective BA. The following BAs were used: cholic acid (CA, Sigma Aldrich, C1129), ursocholic acid (UCA, Avanti, 700229P), taurodeoxycholic acid (TDCA, Avanti, 700250P), taurocholic acid (TCA, Sigma Aldrich, T4009), hyocholic acid (HCA, Avanti, 700159P), ursodesoxycholic acid (UDCA, Sigma Aldrich, U5127), alpha-muricholic acid (α-MCA, Avanti, 700232P), beta-muricholic acid (β-MCA, Sigma Aldrich, SML2372), omega-muricholic acid (ω-MCA, Avanti, 700231P), tauro-alpha-muricholic acid (tauro-α-MCA, Avanti, 700243P), and deoxycholic acid (DCA, Sigma Aldrich, D5670). The secretion buffer was replaced with the respective stimulation buffer, and cells were incubated for 3h in the cell culture incubator. Supernatants were collected, spun down at 900 rcf for 5 min at 4°C, and stored at −80°C. Active GLP-1 was measured using the V-PLEX Plus GLP-1 (active) Kit (mesoscale, K1503OG) according to the manufacturer’s protocol. The cell plate was washed with PBS and stored at −80°C for the following protein isolation and total protein determination used for data normalization (see Section 2.5).

### 2.9 Statistical analysis and quantification

Results are presented as mean ± SEM. Statistical analyses other than ANCOVA were performed with GraphPad Prism 9 and 10. An unpaired two-tailed *t*-test was done for comparison of two groups. An ordinary one-way analysis of variance (ANOVA) was applied for comparisons of three or more groups. Two-way ANOVA was applied for comparisons of two groups across multiple parameters. The ANCOVA analysis was done using the Energy Expenditure Analysis page^1^ provided by NIDDK Mouse Metabolic Phenotyping Centers^2^. Statistical significance is indicated as *p < 0.05, **p < 0.01, ***p < 0.001, and ****p < 0.0001.

## 3 Results

### 3.1 Increased glucose tolerance and cold sensitivity upon gut microbiota-depletion is independent of UCP1^+^ cells

**Figure 1:**
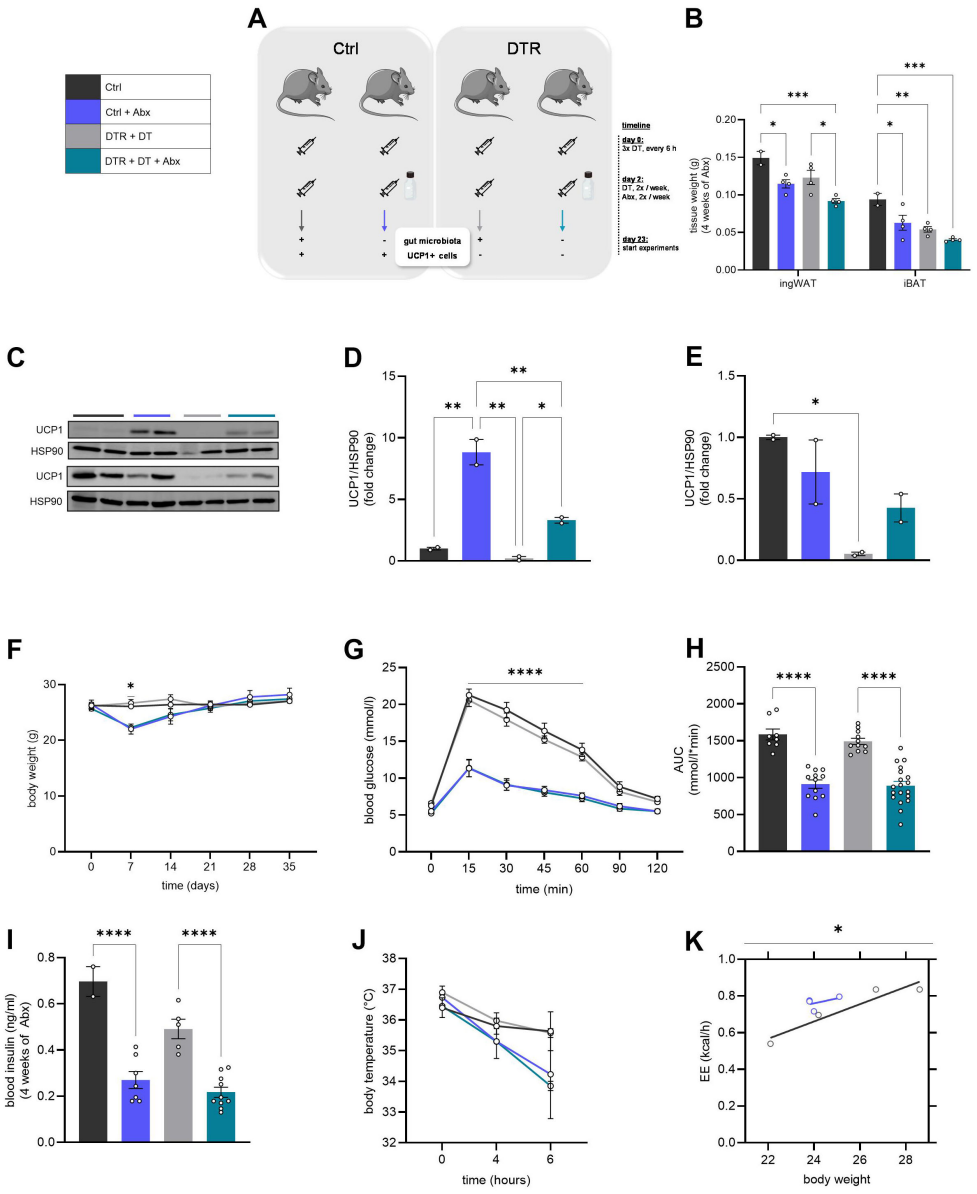
Gut microbiota depletion leads to improved glucose tolerance at room temperature independent of UCP1^+^ cells and increases cold sensitivity. **(A)** Experimental mouse model setup. **(B)** Fat mass of ingWAT and iBAT (Ctrl: *n* = 2; Ctrl + Abx: *n* = 4; DTR + DT: *n* = 4; DTR + DT + Abx: *n* = 4). **(C–E)** Western blot **(C)** of UCP1 in ingWAT (top) and iBAT (bottom) of Ucp1DTR mice (*n* = 2/group) and quantification of protein in ingWAT **(D)** and iBAT **(E)**. **(F)** Body weight development over the course of experiment (Ctrl: *n* = 5; Ctrl + Abx: *n* = 7; DTR + DT: *n* = 6; DTR + DT + Abx; *n* = 12). **(G–H)** Intraperitoneal glucose tolerance test **(G)** and corresponding AUC **(H)** (Ctrl: *n* = 8; Ctrl + Abx: *n* = 12; DTR + DT: *n* = 11; DTR + DT + Abx: *n* = 19). **(I)** Fasted plasma insulin levels (Ctrl: *n* = 2; Ctrl + Abx: *n* = 7; DTR + DT: *n* = 5; DTR + DT + Abx: *n* = 10). **(J)** Rectal body temperature in mice exposed to 8°C during fasting (Ctrl: *n* = 3; Ctrl + Abx: *n* = 3; DTR + DT: *n* = 4; DTR + DT + Abx: *n* = 2). **(K)** Energy expenditure in mice exposed to 8°C for 5 days (*n* = 4/group). Results are presented as average ± SEM. Statistical significance is indicated as *p < 0.05, **p < 0.01, ***p < 0.001, ****p < 0.0001. An ordinary one-way analysis of variance (ANOVA) was applied for **(B, D, E, H, I)**. Two-way ANOVA was applied for **(F, G, J)**. Energy expenditure **(K)** was analyzed using ANCOVA with body weight as covariate.

To study the contribution of brown and beige adipocytes to enhanced glucose tolerance upon gut microbiota deletion, we used the Ucp1Dtr2Gfp mouse line (Ucp1DTR), which allows for transient ablation of UCP1^+^ cells upon diphtheria toxin (DT) injections as described previously (21) (**Figure 1A**). Wild-type (WT) mice served as control for the inducible Ucp1DTR mouse model, and all animals received subcutaneous (sc.) DT injections twice per week. This ensured the ablation of UCP1^+^ cells in Ucp1DTR mice, while WT mice retained brown and brite adipocytes. Within both genotypes, one group received an antibiotic cocktail (Abx) administered in the drinking water for 3 weeks before starting experiments to deplete the gut microbiota (Ctrl + Abx, DTR + DT + Abx), while the other groups (Ctrl, DTR + DT) retained their gut microbiome. Gut microbiota depletion resulted in reduced adipose tissue weight (**Figure 1B**), which is in line with the reported reduced adiposity (12, 13). Furthermore, UCP1^+^ cell depletion resulted in decreased depot mass, as expected (**Figure 1B**). For confirmation, we measured UCP1 protein expression in both ingWAT and iBAT. Abx treatment indeed increased UCP1 protein expression in ingWAT, however, injection of DT significantly reduced UCP1 protein levels in both ingWAT and iBAT in Ucp1DTR mice at room temperature (**Figures 1C–E**). Mice treated with Abx experienced a significant body weight loss within the first seven days of treatment but recovered and even gained weight compared to their controls at room temperature (RT; **Figure 1F**), which aligns with previous studies (22). Interestingly, mice at either thermoneutrality (TN; **Supplementary Figure S1A**) or cold exposure (CE; **Supplementary Figure S1B**) showed increased body weight after 4 weeks of Abx treatment. This finding is likely to be explained by the increased food intake that was observed in mice treated with Abx for 4 weeks at CE (**Supplementary Figure S1C**). Following the 3 weeks of Abx treatment, we conducted an intraperitoneal glucose tolerance test (ipGTT). Surprisingly, the significant improvement in glucose tolerance was independent of UCP1^+^ cell presence in all condition tested – at RT (**Figures 1G, H**), at TN (**Supplementary Figures S1D**, **S1E**) and at CE (**Supplementary Figures S1F**, **S1G**). Blood insulin levels were significantly decreased in mice upon antibiotic-induced gut microbiota depletion at RT (**Figure 1I**), but not at TN (**Supplementary Figure S1H**) and CE (**Supplementary Figure S1I**). These data demonstrate that gut microbiota depletion-dependent improved glucose metabolism is independent of UCP1^+^ cells.

To assess the potential contribution of gut microbiota to thermogenesis independent of the thermogenic role of UCP1^+^ cells, we compared rectal body temperature in all experimental groups (Ctrl, Ctrl + Abx, DTR + DT, DTR + DT + Abx) during fasting at room temperature (0h) and cold exposure (4h and 6h). Mice with antibiotic-induced gut microbiota depletion experienced a stronger decrease in rectal body temperature during cold exposure than their controls (**Figure 1J**). This body temperature drop was even more pronounced in mice lacking both gut microbiota and UCP1^+^ cells (**Figure 1J**), suggesting that gut microbiota contributes to thermogenesis independently of UCP1^+^ cells. Surprisingly, energy expenditure was improved in Abx-treated mice compared to WT controls upon 5 days of CE (**Figure 1K**). Due to severity constraints, energy expenditure was not measured in mice with both microbiota depletion and UCP1^+^ cell ablation, resulting in the exclusion of these experimental groups from analysis. Altogether, these findings suggest that gut microbiota plays a significant role in thermogenesis, influencing both cold sensitivity and energy expenditure.

### 3.2 GLP-1 signaling improves glucose tolerance upon gut microbiota-depletion

**Figure 2:**
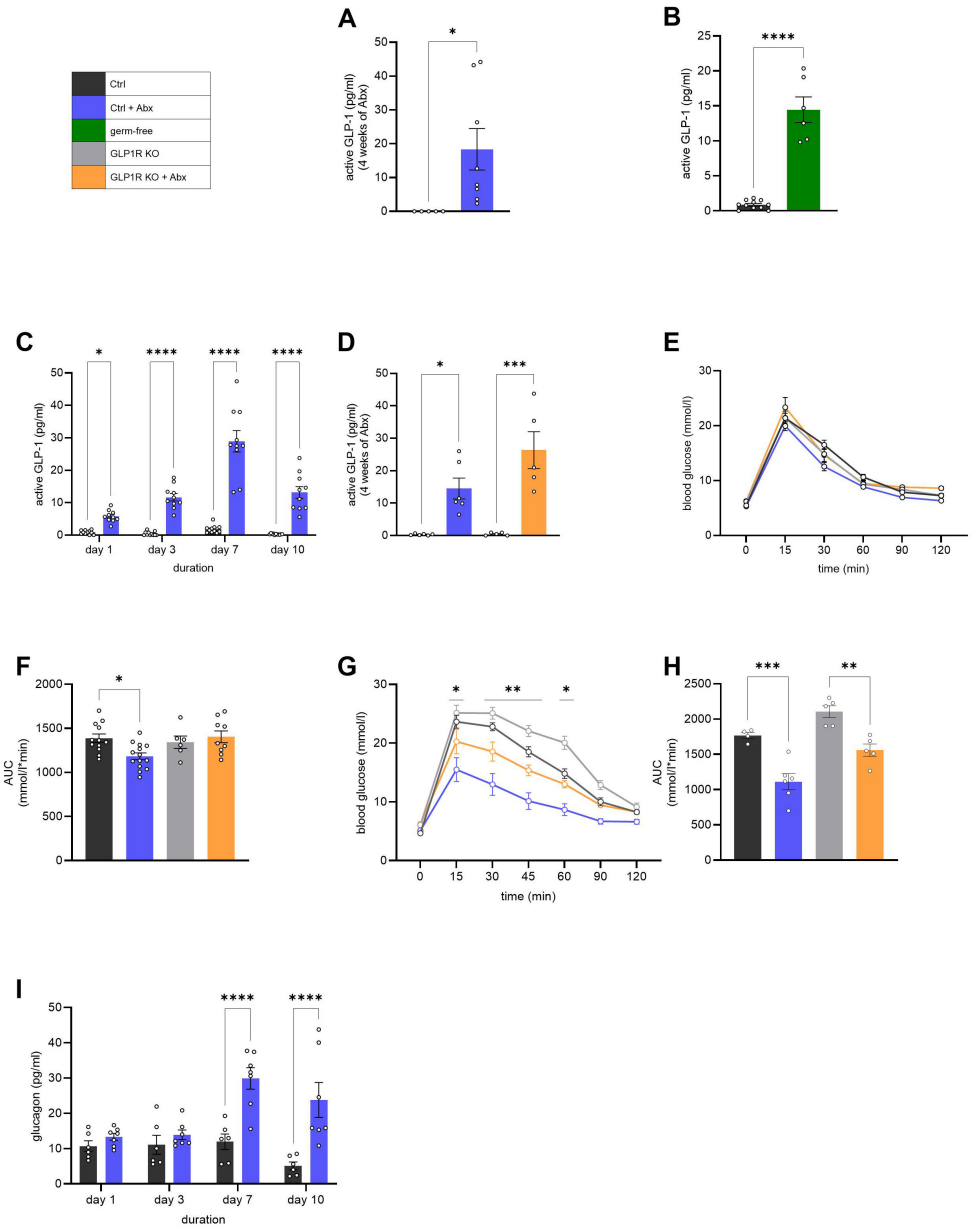
GLP-1 is the key driver of gut microbiota depletion-dependent glucose tolerance improvement. **(A, B)** Fasted active plasma GLP-1 levels in mice upon antibiotic-induced gut microbiota depletion **(A)** (Ctrl: *n* = 5; Ctrl + Abx: *n* = 8) and in germ-free mice **(B)** (Ctrl: *n* = 11; GF: *n* = 6). **(C)** Changes in fasted active plasma GLP-1 levels upon antibiotic treatment (Ctrl: *n* = 11; Ctrl + Abx: *n* = 10). **(D)** Fasted active plasma GLP-1 levels in GLP1R KO mice (Ctrl: *n* = 6; Ctrl + Abx: *n* = 6; GLP1R KO: *n* = 5; GLP1R KO + Abx: *n* = 5). **(E, F)** Oral glucose tolerance test **(E)** and corresponding AUC **(F)** (Ctrl: *n* = 12; Ctrl + Abx: *n* = 14; GLP1R KO: *n* = 6; GLP1R KO + Abx: *n* = 9). **(G**, **H)** Intraperitoneal glucose tolerance test **(G)** and corresponding AUC **(H)** (Ctrl: *n* = 4; Ctrl + Abx: *n* = 6; GLP1R KO: *n* = 5; GLP1R KO + Abx: *n* = 5). **(I)** Changes in fasted plasma glucagon levels upon antibiotic treatment (Ctrl: *n* = 6; Ctrl + Abx: *n* = 7). Results are presented as average ± SEM. Statistical significance is indicated as *p < 0.05, **p < 0.01, ***p < 0.001, ****p < 0.0001. Unpaired two-tailed *t*-test was applied for **(A, B)**. Two-way analysis of variance (ANOVA) was applied for **(C**, **E**, **G**, **I)**. Ordinary one-way ANOVA was applied for (**D**, **F**, **H**).

Next, we aimed to identify the main driver of gut microbiota depletion-dependent improved glucose metabolism. It has been shown previously (7, 15, 23, 24) that active GLP-1 is significantly improved upon antibiotic-induced gut microbiota depletion. Indeed, mice treated with Abx for 4 weeks showed significant elevation of baseline active GLP-1 in plasma compared to control mice in a fasted state (**Figure 2A**). Studies have shown that antibiotic-induced depletion does not fully erase the gut microbiota but significantly reduces its abundance and alters its composition (7, 22, 25, 26). To further confirm that the increase in active GLP-1 is independent of the remaining gut microbiota, we measured active GLP-1 in GF mice. Notably, active GLP-1 was also significantly elevated in fasted GF mice compared to controls (**Figure 2B**). These results suggest that the elevation of active GLP-1 is driven by the absence of gut microbiota, independent of any remaining microbial presence. To identify the optimal Abx treatment duration, we performed a treatment time course. Abx was given to the treatment group, and plasma was collected on days 1, 3, 7, and 10. Surprisingly, even one day of Abx treatment was sufficient to yield a significant elevation of active GLP-1 in the plasma compared to control mice and increased further until day 7 before stabilizing at a level already seen on day 3 (**Figure 2C**). Thus, we performed the following experiments after at least 3 days of antibiotic treatment. GLP-1 is primarily secreted in response to nutrient ingestion and plays a key role in the incretin effect. To assess the role of GLP-1 in improving glucose tolerance following antibiotic-mediated gut microbiota depletion, we first performed an oral glucose tolerance test (oGTT) in control and whole-body GLP-1 receptor knockout (GLP1R KO) mice with or without antibiotic-induced gut microbiota depletion. These mice also exhibited significantly elevated active GLP-1 levels in a fasted state after Abx-mediated gut microbiota depletion (**Figure 2D**). While gut microbiota depletion in control mice resulted in improved glucose tolerance, this effect was fully reversed upon a whole-body knockdown of the GLP-1 receptor (**Figures 2E, F**), suggesting that GLP-1 signaling is essential for the observed glucose tolerance improvement. Given the elevated basal levels of GLP-1 following gut microbiota depletion independent of nutrient ingestion, we hypothesized that the observed improvement in glucose tolerance would also be abolished in GLP1R KO mice during an ipGTT. Surprisingly, however, the improvement in glucose tolerance was still observed in GLP1R KO mice following antibiotic treatment (**Figures 2G, H**). These results indicate that there is a GLP1R-independent mechanism contributing to enhanced peripheral glucose homeostasis upon gut microbiota depletion.

A possible mediator is glucagon, which is known to be regulated by GLP-1 (27, 28). Thus, we measured plasma glucagon levels at different timepoints during antibiotic administration and measured *Gcgr* gene expression in liver and ingWAT. *Gcgr* gene expression was not changed in either ingWAT or liver (**Supplementary Figure S2A–S2F**). Surprisingly, fasting plasma glucagon levels were increased 7 days upon antibiotic treatment compared to control mice (**Figure 2I**). As this effect is not observed at the earlier timepoints, we conclude that glucagon is not mediating the secondary effects on glucose metabolism improvement.

### 3.3 HCA and UDCA are regulated by the gut microbiome and act as potential GLP-1 secretion inhibitors

**Figure 3:**
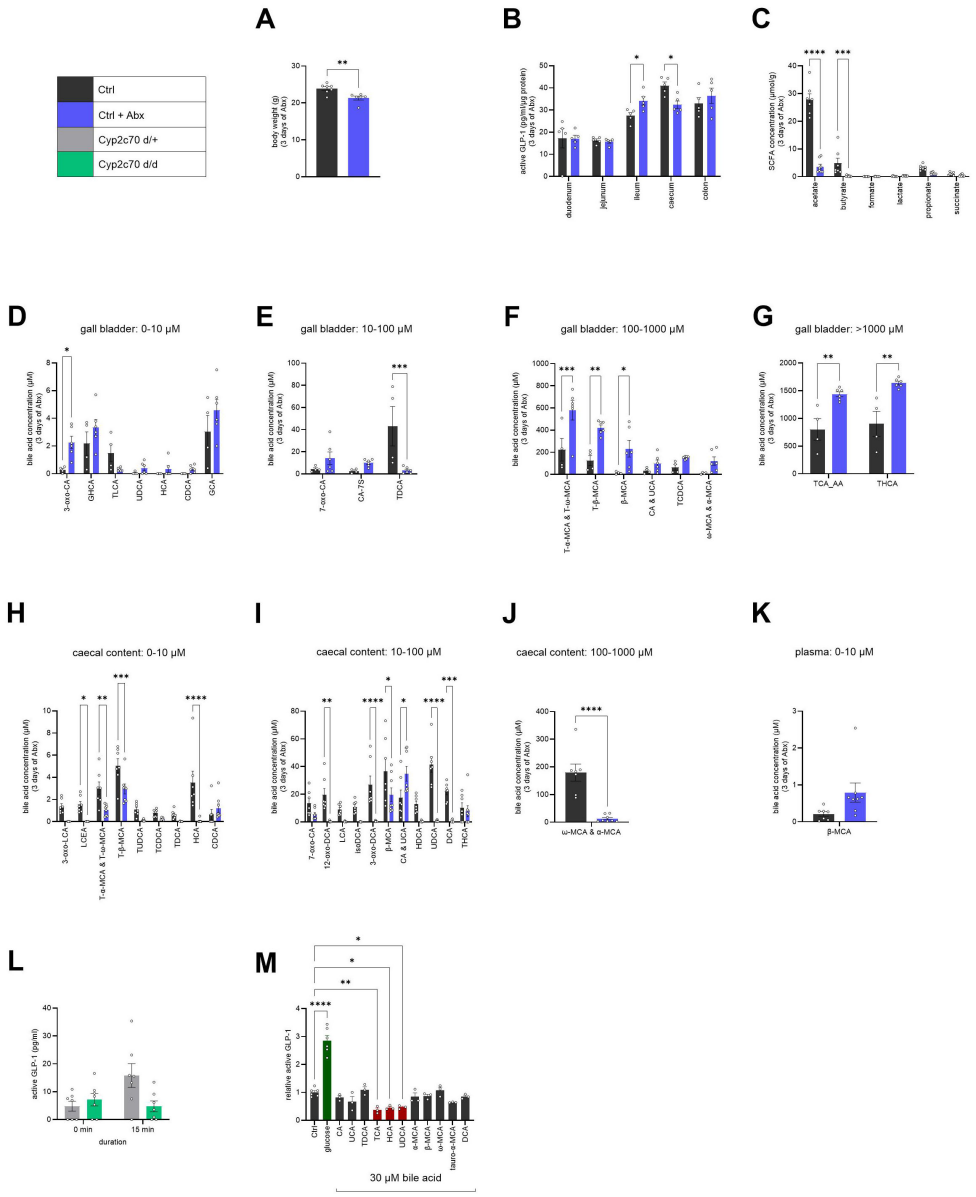
Antibiotic-induced gut microbiota depletion alters bile acid pool and 3 bile acids inhibit basal GLP-1 secretion *in vitro*. **(A)** Body weight upon 3 days of antibiotic treatment (Ctrl: *n* = 7; Ctrl + Abx: *n* = 6). **(B)** Active GLP-1 levels in the different intestinal parts (*n* = 5/group). **(C)** Levels of different short-chain fatty acid in feces (Ctrl: *n* = 7; Ctrl + Abx: *n* = 8). **(D–K)** Changes in bile acids upon 3 days of antibiotic treatment in gall bladder **(D–G)**, feces **(H–J)** and plasma **(K)** (Ctrl: *n* = 4; Ctrl + Abx: *n* = 6). **(L)** Active plasma GLP-1 levels in a fasted state and after gavage of mixed meal (n = 7/group). **(M)** GLP-1 secretion assay in STC-1 cells stimulated with 30 µM bile acids or glucose for 3 h (Ctrl/glucose: *n* = 6/group; bile acids: *n* = 3/group). Results are presented as average ± SEM. Statistical significance is indicated as *p < 0.05, **p < 0.01, ***p < 0.001, ****p < 0.0001. Unpaired two-tailed *t*-test was applied for **(A, J, K)**. Two-way analysis of variance (ANOVA) was applied for **(B–I, L)**. Ordinary one-way ANOVA was applied for **(M)**.

First, we aimed to determine the main source of increased GLP-1 secretion. WT mice were treated with an antibiotic cocktail for 3 days. As expected, gut microbiota-depleted mice showed decreased body weight compared to the control mice (**Figure 3A**). We measured active GLP-1 levels in the different intestinal parts, namely, duodenum, jejunum, ileum, caecum, and colon. Intestinal tissue weight was not different; however, a tendency of increased caecum weight was observed compared to control mice (**Supplementary Figure S3A**). This is in line with previous reports that showed that antibiotic-induced gut microbiota depletion leads to a highly enlarged caecum (29). Significantly increased active GLP-1 was measured in the ileum upon Abx-dependent gut microbiota depletion compared to control mice (**Figure 3B**). Depletion of the gut microbiome with Abx modulates the presence of luminal secondary metabolites such as SCFAs and BAs, which consequently affect gut signaling and potentially whole-body glucose metabolism. Thus, we next examined the SCFA composition, key gut-derived metabolites modulated by the composition of the gut microbiome and potential stimulators of GLP-1 release (30, 31). We collected the feces of mice treated with antibiotics for 3 days and analyzed the SCFAs. In line with previous studies, we found that acetate and butyrate were significantly reduced upon Abx-induced gut microbiota depletion compared to control mice (**Figure 3C**). As both are known to act as GLP-1 stimulators (30, 31), we conclude that while gut microbiota depletion leads to a significantly altered SCFA composition, this change does not drive the increase in active GLP-1. Given that antibiotics not only deplete the gut microbiota but also damage the gut barrier and overall lead to gut dysbiosis (32, 33), we investigated whether lipopolysaccharide (LPS) levels changed in the intestinal regions that exhibited altered GLP-1 secretion following 3 days of antibiotic-induced gut microbiota depletion, as well as in plasma (**Supplementary Figures S3B**, **S3C**). However, we observed no significant changes in LPS levels either in plasma (**Supplementary Figure S3B**) or in the ileum, caecum or colon (**Supplementary Figure S3C**). This is in line with a previous study, which found that gut-derived LPS is not essential for the improvement in glucose tolerance observed after gut microbiota depletion (13). BAs are modified by the gut microbiota and known to act as GLP-1 secretion regulators, thereby modulating glucose metabolism and insulin sensitivity (23, 34–39). Therefore, we aimed to identify the BAs that are regulated upon antibiotic-induced gut microbiota depletion. BA profiling (**Table 2**) was conducted in the bile, feces, and plasma of mice treated with Abx for 3 days compared to control mice (**Figures 3D–K** and **Supplementary Figures S3D–S3F**). Various BAs were changed in concentration upon antibiotic-induced gut microbiota depletion as shown in (**Figures 3D–K**). We first focused on the muricholic acids (MCAs) ω-MCA and α-MCA, which were most significantly regulated amongst all BAs in feces (**Figure 3J**), as potential GLP-1 modulators. We used the Cyp2c70 knockout mouse model, which lacks the Cyp2c70 enzyme responsible for the synthesis of MCAs, leading to an altered, more human-like BA pool (40–43). Plasma was collected in fasted mice and 15 min following oral mixed meal ingestion. Interestingly, basal active GLP-1 was not different, but active GLP-1 secretion upon meal ingestion was blunted in mice lacking MCAs (**Figure 3L**). This data indicates that MCAs play a role in postprandial active GLP-1 release in mice but demonstrates that MCAs do not regulate basal active GLP-1.

**Table 2:** List of bile acids measured with full names.

Bile acid abbreviation	Full name
CA	Cholic acid
CDCA	Chenodeoxycholic acid
DCA	Deoxycholic acid
GCA	Glycocholic acid
GCDCA	Glycochenodeoxycholic acid
GDCA	Glycodeoxycholic acid
GHDCA	Glyco-hyodeoxycholic acid
GLCA	Glycolithocholic acid
GUDCA	Glycoursodeoxycholic acid
LCA	Lithocholic acid
HDCA	Hyodeoxycholic acid
TCA	Taurocholic acid
TCDCA	Taurochenodeoxycholic acid
TDCA	Taurodeoxycholic acid
THCA	Taurohyocholic acid
THDCA	Taurohyodeoxycholic acid
TLCA	Taurolithocholic acid
TUDCA	Tauroursodeoxycholic acid
T-α-MCA	Tauro-α-Muricholic acid
T-β-MCA	Tauro-β-Muricholic acid
T-ω-MCA	Tauro-ω-Muricholic acid
UDCA	Ursodeoxycholic Acid
α-MCA	α-Muricholic acid
β-MCA	β-Muricholic acid
HCA	Hyocholic acid
ω-MCA	ω-Muricholic acid
iaLCA	isoalloLithocholic acid
3-oxo-LCA	3-oxo-Lithocholic acid
3-oxo-CA	3-oxo-cholic acid
7-oxo-CA	7-oxo-cholic acid
7-oxo-CDCA	7 Keto Lithocholic acid
12-oxo-DCA	12-oxo-Deoxycholic Acid
iso LCA	iso Lithocholic acid
3-oxo-DCA	3-oxo-Deoxycholic Acid
iso DCA	iso Deoxycholic Acid
24-norUDCA	norUDCA
5-CholA	5-Cholenic acid-3β-ol
LCEA	Lithocholenic acid
23NorDCA	23-Nordeoxycholic acid
UCA	Ursocholic acid
GUCA	Glycoursocholanic acid
LCA 3-sulfate	Lithocholic acid 3-Sulfate
GDHCA	Glycodehydrocholic acid
GHCA	Glycohyocholic acid
GLCA-3_Sul	Glycolithocholic Acid-3-Sulfate
CA-7-Sul	Cholic Acid-7-Sulfate
TLCA-3-Sul	Taurolithocholic Acid-3-Sulfate

Next, we sought to examine the effects of all significantly regulated BAs on GLP-1 secretion. Thus, we performed a GLP-1 secretion assay in STC-1 cells, stimulating starved cells with the different BAs at a concentration of 30 µM. We were able to identify three inhibitors of GLP-1 secretion, namely taurocholic acid (TCA), hyocholic acid (HCA), and ursodesoxycholic acid (UDCA; **Figure 3M**). As shown in our BA profiling data (**Figures 3H, I**), HCA and UDCA were significantly reduced in feces, while TCA was increased in gall bladder (**Figure 3G**). In the BA pool of GF mice, which have elevated basal active GLP-1 (as shown in **Figure 2B**), it has been shown that taurine-conjugated BAs are present while secondary BAs such as HCA and UDCA are likely reduced due to the absence of gut microbiota (23, 44). Hence, we propose that HCA and UDCA may be potential inhibitors of basal GLP-1 secretion, and their absence following antibiotic-induced gut microbiota depletion could drive the observed improvement in glucose metabolism by relieving the inhibition of basal GLP-1 secretion.

## 4 Discussion

It has been previously suggested that increased UCP1 in WAT contributes to improved glucose tolerance following gut microbiota depletion (12), partially supported by findings showing increased glucose uptake in BAT and the caecum, although without a corresponding impact on thermogenesis (20). In contrast, other studies demonstrating that gut microbiota depletion leads to decreased body temperature and reduced UCP1 levels questioned this assumption (16–18). In line with these latter findings, we confirmed that glucose tolerance improvement upon gut microbiota depletion is independent of UCP1^+^ cells, as demonstrated using an inducible Ucp1DTR mouse model at RT, TN, and CE. Additionally, gut microbiota depletion increased cold sensitivity independent of UCP1^+^ cells. This suggests the existence of compensatory thermogenic pathways that need to be further explored. Interestingly, gut microbiota-depleted Ctrl mice exhibited increased energy expenditure and elevated UCP1 protein levels in ingWAT in these mice may contribute to this effect.

Second, we confirmed that gut microbiota depletion significantly elevates basal active plasma GLP-1 levels in both antibiotic treated and GF mice, consistent with previous findings (15, 23). Notably, this increase occurred as early as 24h post-treatment, suggesting that gut microbiota depletion directly influences GLP-1 secretion. The hypothesized critical role of GLP-1 signaling in driving the observed improvement in glucose tolerance was confirmed by the restoration of glucose tolerance improvement in GLP1R KO mice during an oGTT. Surprisingly, it was reported that exendin-9, a GLP-1 receptor antagonist, failed to eliminate the improvement in glucose tolerance during the oGTT (15). It was recently reported that GLP-1 secretion occurs also apically into the intestinal lumen of the human colon (45). This is supported by the demonstrated GLP1R expression in human colonic enterocytes, suggesting paracrine GLP-1 signaling, and alterations in colonic energy metabolism due to GLP1R signaling (45–48). This could explain the failure of intraperitoneally administered exendin-9 to inhibit local GLP-1 effects, whereas a whole-body GLP1R KO eliminated the improvement in glucose tolerance. Surprisingly, the improvement in glucose tolerance persisted in GLP1R KO mice during an ipGTT, suggesting that GLP-1 may influence peripheral glucose metabolism via GLP1R-independent pathways. Growing evidence indicates that GLP-1 or its metabolite GLP-1 (9–36) may signal through alternative receptors, such as glucagon receptor (Gcgr) (49–54). However, we observed no change in *Gcgr* mRNA expression in either ingWAT or liver, and fasting plasma glucagon levels were increased after 7 days of antibiotic treatment, suggesting the involvement of additional, yet unidentified pathways.

We show that elevated GLP-1 secretion likely originates from the ileum, as suggested previously (23). LPS, a potential mediator, is sensed by enteroendocrine L cells via TLR4 receptors following gut barrier disruption (55, 56). However, no change in LPS levels was observed in GLP-1 secreting gut regions or plasma, ruling out LPS as the cause. BAs, regulated by gut microbiota, also play a key role in glucose metabolism (14, 15, 57, 58). After Abx-mediated gut microbiota depletion, we identified several regulated BAs, including ω-MCA and α-MCA, which have been reported (15). Interestingly, when we assessed GLP-1 secretion in a Cyp2c70 KO mouse model lacking MCAs (41–43), postprandial GLP-1 secretion was blunted, supporting previous findings that ω-MCA stimulates GLP-1 secretion in primary ileal cells (23). *In vitro* experiments identified TCA, UDCA, and HCA as BA inhibitors of GLP-1 secretion. Since GF mice, which show increased basal GLP-1 secretion, lack secondary BAs but contain taurine-conjugated BAs (59, 60), we exclude TCA as potential driver of the observed phenotype. UDCA has been shown to stimulate GLP-1 secretion in previous studies (61–63). Moreover, it has been shown that TGR5 and FXR ablation do not affect basal GLP-1 levels (23). This aligns with our data, showing the critical role of ω-MCA in postprandial GLP-1 secretion in the Cyp2c70 KO mouse model and additionally proposing HCA as the potential inhibitor of basal GLP-1 secretion, probably independent of TGR5 and FXR signaling.

Together, these findings refine and expand the current understanding of how gut microbiota depletion modulates glucose metabolism. Using an inducible Ucp1DTR mouse model, we demonstrate that the improvement in glucose tolerance occurs independently of UCP1^+^ cells. Additionally, by using a whole-body GLP1R knockout model, we provide direct genetic evidence that the improvement in oral glucose tolerance depends fully on GLP-1 receptor signaling, thereby overcoming the limitations of previous studies based on pharmacological inhibition. Furthermore, our data adds mechanistic insights by distinguishing between the regulation of basal and postprandial GLP-1 secretion: while ω-MCA promotes nutrient-induced GLP-1 release, we identify UDCA and HCA as potential inhibitors of basal secretion *in vitro*, supported through Cyp2c70 knockout and STC-1 cell experiments.

These observations suggest that gut microbiota-driven alterations in BA composition fine-tune both basal and postprandial GLP-1 secretion. Future *in vivo* studies testing administration of these BAs could provide valuable confirmation of their physiological relevance.

### 4.1 Limitations

While our study provides new insights into how gut microbiota depletion modulates glucose metabolism through GLP-1 signaling, some limitations should be acknowledged.

First, representative Western blots of UCP1 protein expression were obtained from two biological replicates per group. It should be noted however that the reliability efficiency of the Ucp1DTR mouse model has been previously validated (21).

Second, we did not directly quantify microbial load by 16S rRNA sequencing or bacterial qPCR. However, we followed a previously published protocol, which demonstrated that this antibiotic regimen efficiently depletes gut bacteria (12). Moreover, consistent physiological indicators of microbiota depletion, including enlarged caecum and reduced fecal SCFA levels, together with prior validation of the same antibiotic cocktail, strongly support the effectiveness of the treatment.

Third, while we demonstrate a GLP-1–dependent improvement in oral glucose tolerance, our data indicates the involvement of additional pathways beyond canonical GLP1R and GCGR signaling, which remains yet to be determined.

Finally, while bile acid-induced GLP-1 secretion has often been linked to TGR5- or FXR-dependent signaling, basal GLP-1 secretion regulation may be governed by distinct mechanisms, such as modulation of ion channels, other GPCRs expressed in L cells, or changes in bile acid transporters. Further studies will be required to define these mechanisms.

## Data Availability

The raw data supporting the conclusions of this article will be made available by the authors, without undue reservation.

## Footnotes

1 http://www.mmpc.org/shared/regression.aspx.

2 MMPC, www.mmpc.org.
