# Supplementary material for: Antibiotic-induced gut microbiota depletion enhances glucose tolerance linked to GLP-1 signaling
Source: Front Endocrinol (Lausanne). 2025 Nov 27;16:1684155. doi: 10.3389/fendo.2025.1684155 (PMC12695531; doi:10.3389/fendo.2025.1684155)
Supplement: Supplementary file 1 [file DataSheet1.docx]

Antibiotic-induced gut microbiota depletion enhances glucose tolerance linked to GLP-1 signaling

Supplementary Material

Alexandra Kellenberger^1^, Revati Sumukh Dewal^1^, Alice de Wouters d’Oplinter^2^, Andreas Sichert^2^, Markus Heine^3^, Marceline M. Fuh^3^, Emma Slack^2^, Tenagne Delessa Challa^1*^ & Christian Wolfrum^1*^

^1^Laboratory of Translational Nutrition Biology, Institute of Food, Nutrition and Health, Department of Health Sciences and Technology, ETH Zurich, Schwerzenbach, Switzerland.

^2^Laboratory for Mucosal Immunology, Institute of Food, Nutrition and Health, Department of Health Sciences and Technology, ETH Zurich, Zurich, Switzerland.

^3^Department of Biochemistry and Molecular Cell Biology, University Medical Center Hamburg-Eppendorf, Hamburg, Germany.

*** Correspondence:**Tenagne Delessa Challa
[tenagne.challa@hest.ethz.ch](mailto:tenagne.challa@hest.ethz.ch)

Christian Wolfrum
[christian-wolfrum@ethz.ch](mailto:christian-wolfrum@ethz.ch)

# Supplementary Figures

## Supplementary Figure 1


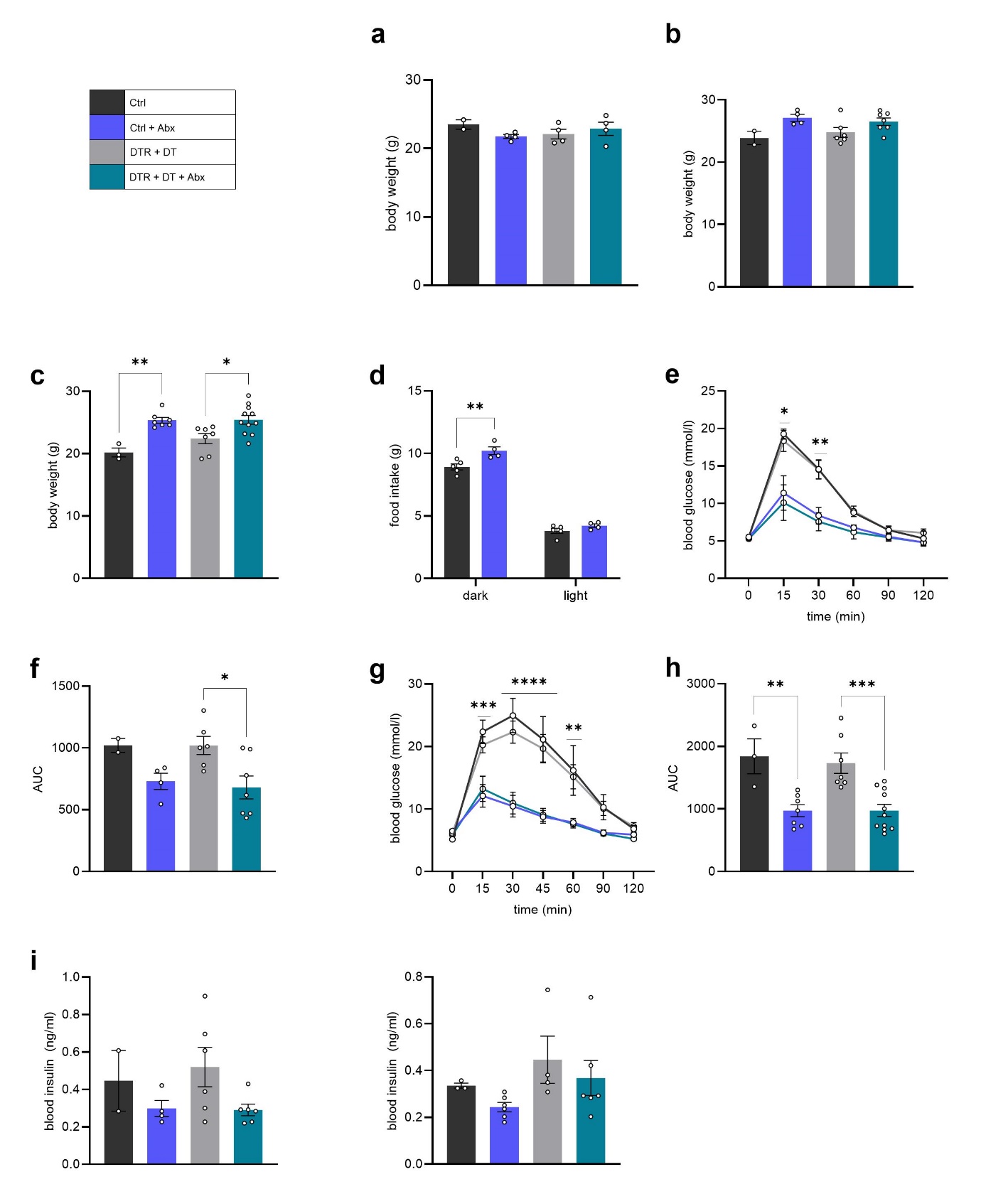


Suppl. Figure 1: **Gut microbiota depletion-dependent improved glucose tolerance is independent of UCP1^+^ cells at thermoneutrality and cold.** (a-b) Body weight change in mice upon antibiotic treatment at thermoneutrality (a; Ctrl: n = 2; Ctrl + Abx: n = 4; DTR + DT: n = 6; DTR + DT + Abx: n = 7) and cold (b; Ctrl: n = 3; Ctrl + Abx: n = 7; DTR + DT: n = 7; DTR + DT + Abx: n = 11). (c) Food intake in mice upon 5 days of cold exposure (Ctrl: n = 5; Ctrl + Abx: n = 4). (d-e) Intraperitoneal glucose tolerance (d) and corresponding AUC (e) in mice at thermoneutrality (Ctrl: n = 2; Ctrl + Abx: n = 4; DTR + DT: n = 6; DTR + DT + Abx: n = 7). (f-g) Intraperitoneal glucose tolerance (f) and corresponding AUC (g) in mice at cold (Ctrl: n = 3; Ctrl + Abx: n = 7; DTR + DT: n = 7; DTR + DT + Abx: n = 11). (h-i) Fasting plasma insulin levels in mice at thermoneutrality (h; Ctrl: n = 2; Ctrl + Abx: n = 4; DTR + DT: n = 6; DTR + DT + Abx: n = 6) and cold (i; Ctrl: n = 3; Ctrl + Abx: n = 6; DTR + DT: n = 4; DTR + DT + Abx: n = 6). Results are presented as average ± SEM. Statistical significance is indicated as *p < 0.05, **p < 0.01, ***p < 0.001, ****p < 0.0001. Ordinary one-way ANOVA was applied for (a, b, e, g, h, i). 2way ANOVA was applied for (c, d, f).

## Supplementary Figure 2


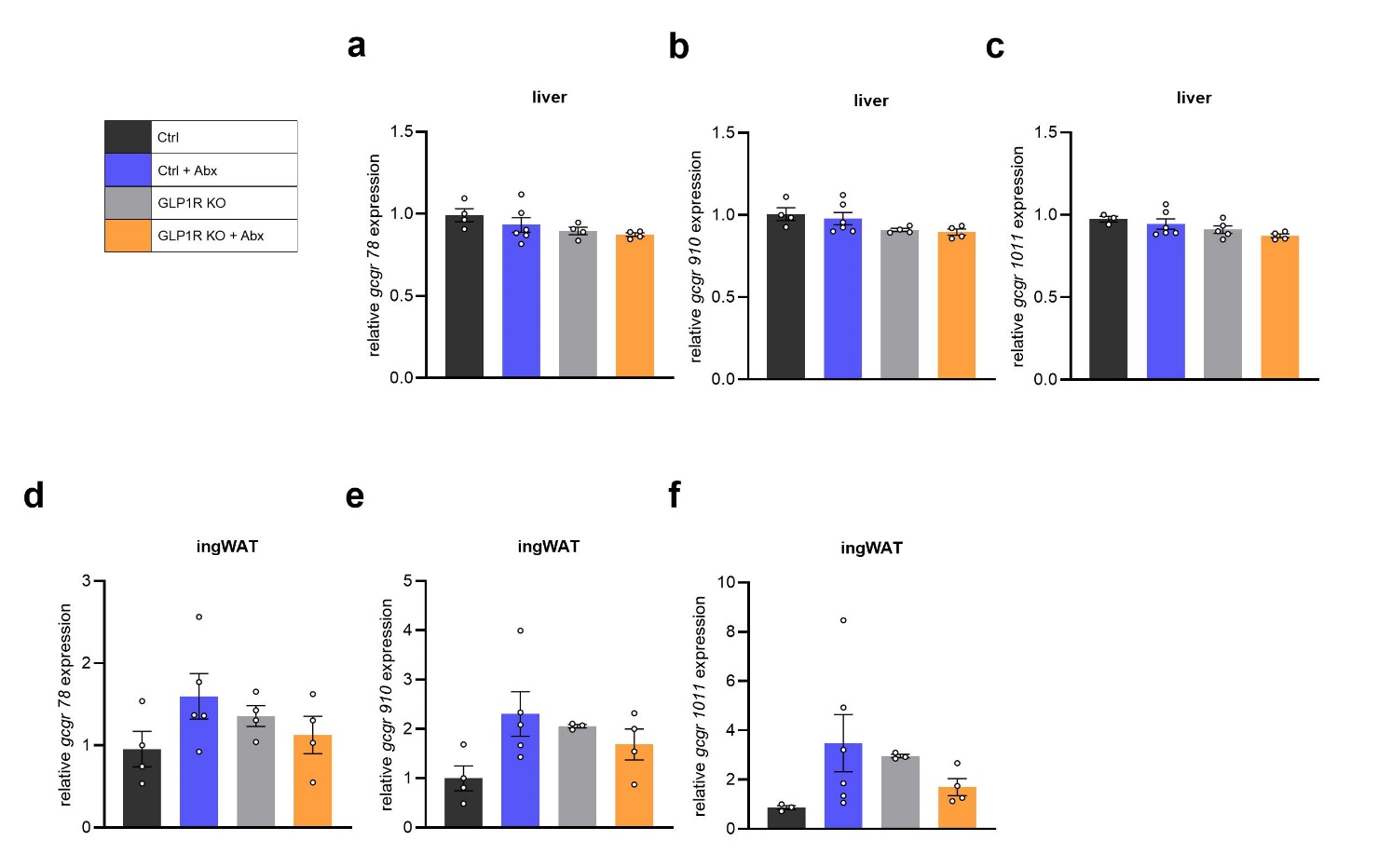


Suppl. Figure 2: Glucagon is not driving the gut microbiota depletion-induced peripheral improvement in glucose tolerance. (a-f) Gene expression in liver (a-c) of gcgr 78 (Ctrl: n = 4; Ctrl + Abx: n = 6; GLP1R KO: n = 4; GLP1R KO + Abx: n = 4), gcgr 910 (Ctrl: n = 4; Ctrl + Abx: n = 6; GLP1R KO: n = 4; GLP1R KO + Abx: n = 4) and gcgr 1011 (Ctrl: n = 3; Ctrl + Abx: n = 6; GLP1R KO: n = 5; GLP1R KO + Abx: n = 4), and in ingWAT (d-f) of gcgr 78 (Ctrl: n = 4; Ctrl + Abx: n = 5; GLP1R KO: n = 4; GLP1R KO + Abx: n = 4), gcgr 910 (Ctrl: n = 4; Ctrl + Abx: n = 5; GLP1R KO: n = 3; GLP1R KO + Abx: n = 4) and gcgr 1011 (Ctrl: n = 3; Ctrl + Abx: n = 6; GLP1R KO: n = 3; GLP1R KO + Abx: n = 4). Results are presented as average ± SEM. Statistical significance is indicated as *p < 0.05, **p < 0.01, ***p < 0.001, ****p < 0.0001. Ordinary one-way ANOVA was applied for all graphs.

## Supplementary Figure 3


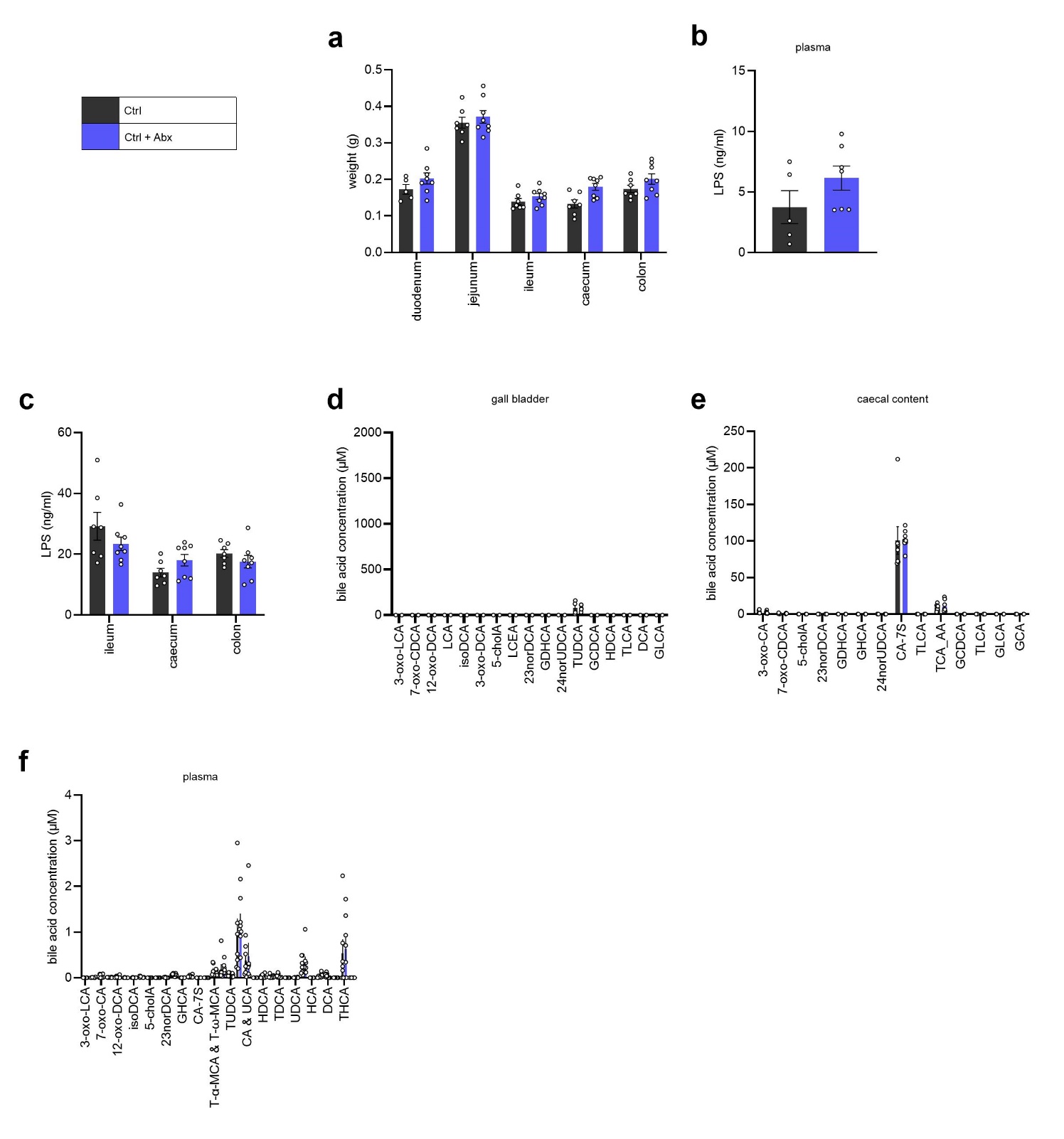


Suppl. Figure 3: LPS is not driving increased GLP-1 secretion. (a) Tissue weight of intestinal parts following 3 days of antibiotic treatment (Ctrl: n = 7; Ctrl + Abx: n = 8). (b-c) Fasted LPS levels in plasma (b) and the intestinal parts ileum, caecum and colon (c) (Ctrl: n = 7; Abx: n = 8). (d-f) Bile acids unregulated upon 3 days of antibiotic treatment in gall bladder (d), feces (e) and plasma (f) (Ctrl: n = 4; Ctrl + Abx: n = 6). Results are presented as average ± SEM. Statistical significance is indicated as *p < 0.05, **p < 0.01, ***p < 0.001, ****p < 0.0001. 2way ANOVA was applied for (a, c, d, e, f). Unpaired two-tailed *t*-test was applied for (b).
