# Supplementary material for: Antibiotic-induced gut microbiota depletion enhances glucose tolerance linked to GLP-1 signaling
Source: Front Endocrinol (Lausanne). 2025 Nov 27;16:1684155. doi: 10.3389/fendo.2025.1684155 (PMC12695531; doi:10.3389/fendo.2025.1684155)
Supplement: Supplementary file 2 [file DataSheet2.docx]

Antibiotic-induced gut microbiota depletion enhances glucose tolerance via GLP-1 signaling

Original Western Blots

Alexandra Kellenberger^1^, Revati Sumukh Dewal^1^, Alice de Wouters d’Oplinter^2^, Andreas Sichert^2^, Markus Heine^3^, Marceline M. Fuh^3^, Emma Slack^2^, Tenagne Delessa Challa^1*^ & Christian Wolfrum^1*^

^1^Laboratory of Translational Nutrition Biology, Institute of Food, Nutrition and Health, Department of Health Sciences and Technology, ETH Zurich, Schwerzenbach, Switzerland.

^2^Laboratory for Mucosal Immunology, Institute of Food, Nutrition and Health, Department of Health Sciences and Technology, ETH Zurich, Zurich, Switzerland.

^3^Department of Biochemistry and Molecular Cell Biology, University Medical Center Hamburg-Eppendorf, Hamburg, Germany.

*** Correspondence:**Tenagne Delessa Challa
[tenagne.challa@hest.ethz.ch](mailto:tenagne.challa@hest.ethz.ch)

Christian Wolfrum
[christian-wolfrum@ethz.ch](mailto:christian-wolfrum@ethz.ch)

# Figure 1C: Original Western Blots

## ingWAT UCP1


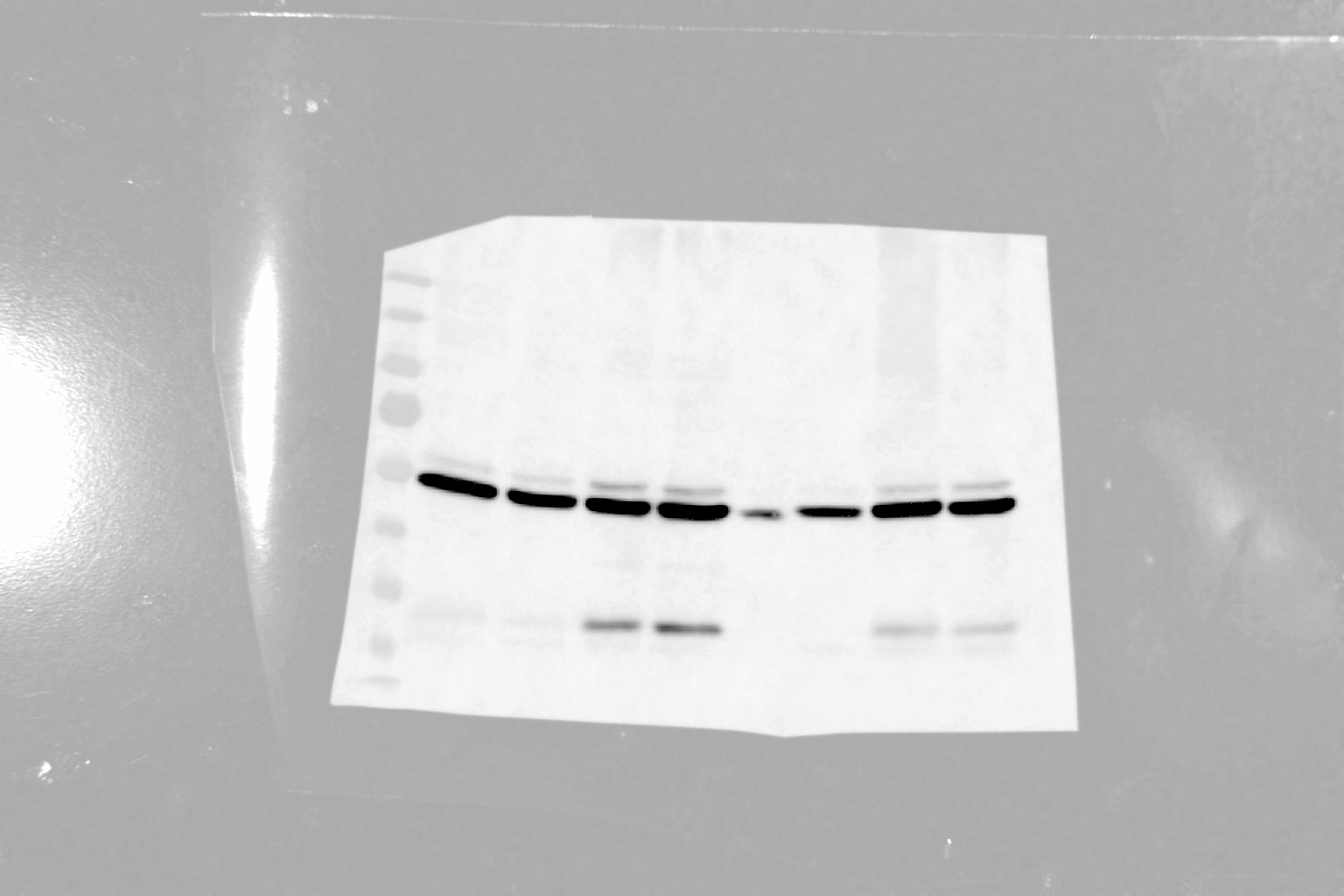


35 kDa

40 kDa

55 kDa

70 kDa

UCP1, 32 kDa

## ingWAT HSP90


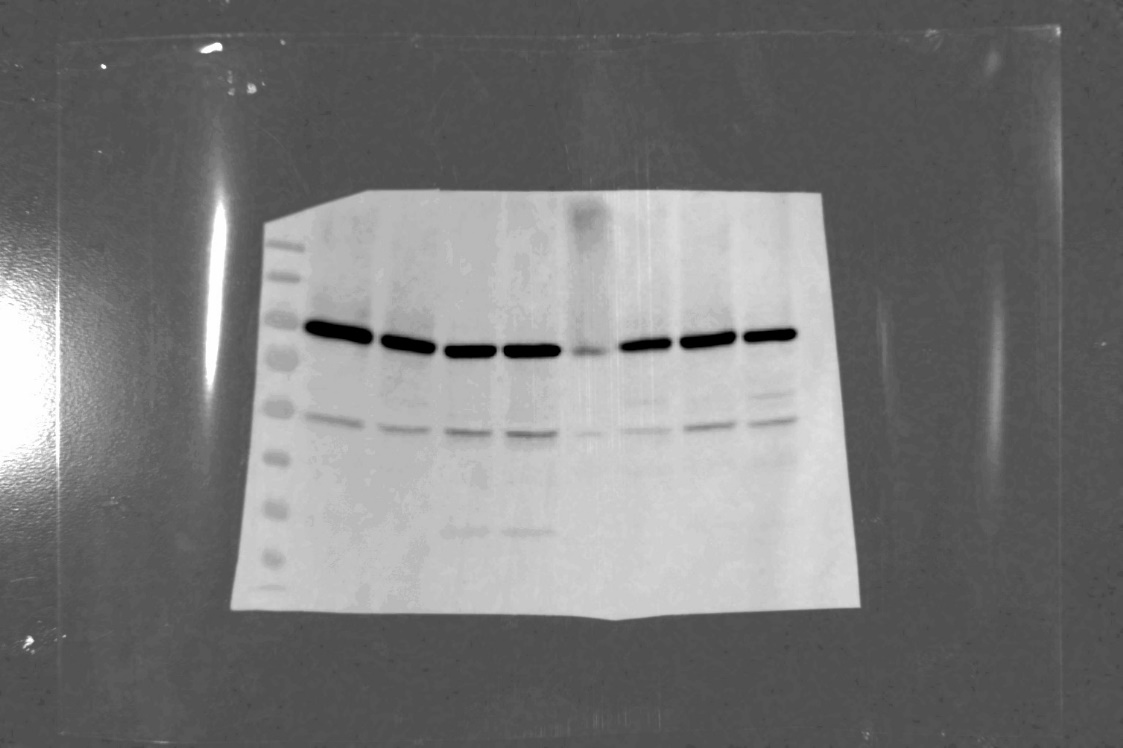


100 kDa

70 kDa

HSP90, 90 kDa

## iBAT UCP1


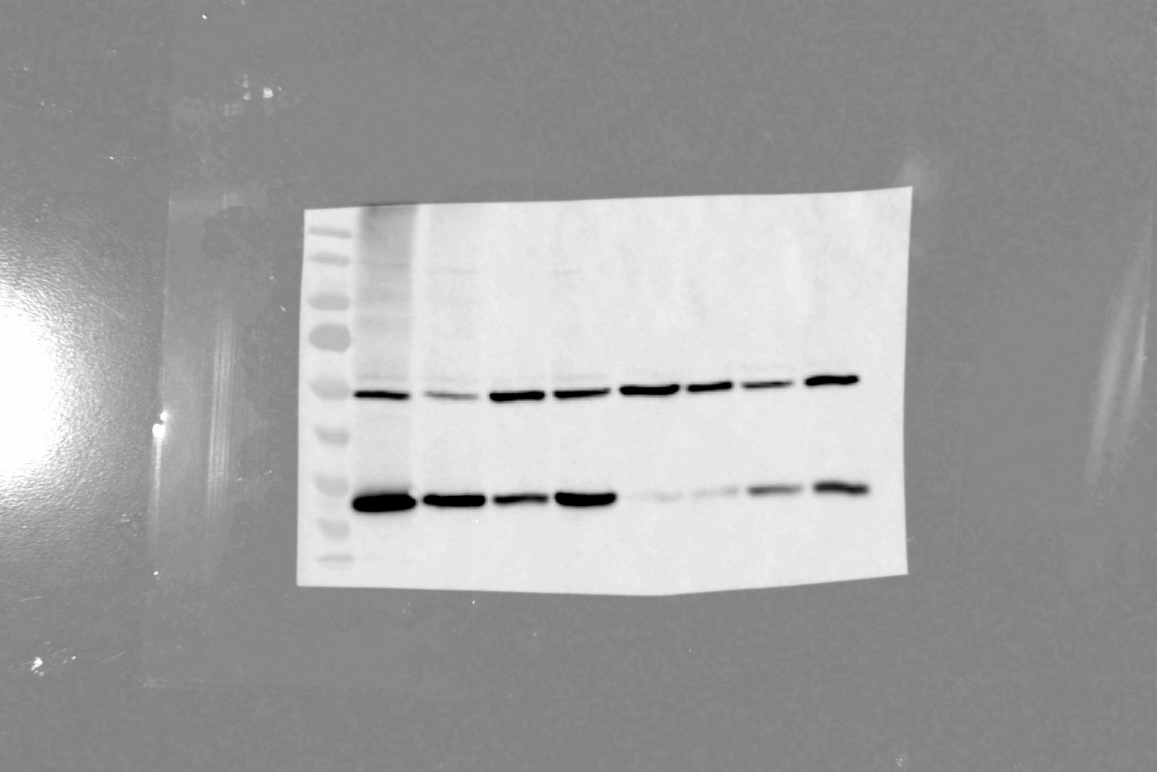


35 kDa

40 kDa

55 kDa

70 kDa

UCP1, 32 kDa

## iBAT HSP90


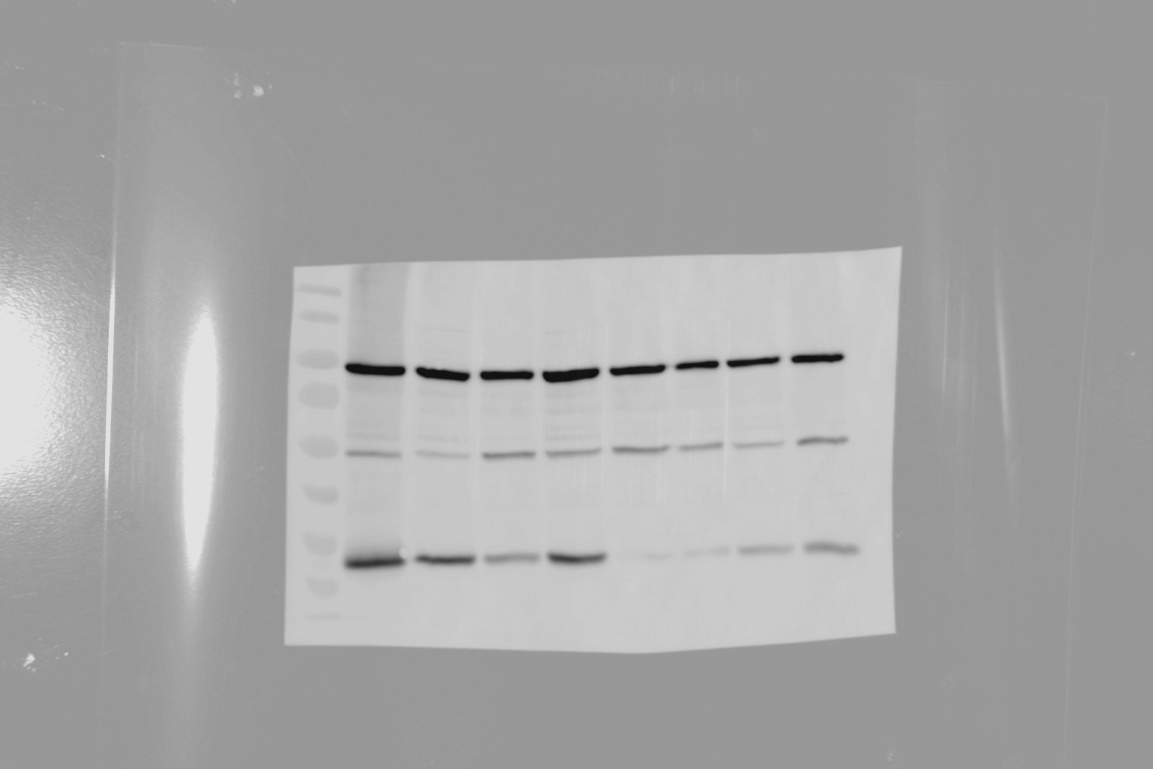


100 kDa

70 kDa

HSP90, 90 kDa
